# Kinetics of Cardiac Remodeling and Fibrosis Biomarkers During an Extreme Mountain Ultramarathon

**DOI:** 10.3389/fcvm.2022.790551

**Published:** 2022-03-07

**Authors:** Caroline Le Goff, Magalie Viallon, Jean-François Kaux, Pierre Andonian, Kevin Moulin, Laurence Seidel, Guido Giardini, Laurent Gergelé, Pierre Croisille, Etienne Cavalier, Gregoire P. Millet

**Affiliations:** ^1^Department of Clinical Chemistry, CHU de Liège, University of Liège, Liège, Belgium; ^2^Department of Radiology, University Hospital of Saint Etienne, Saint-Étienne, France; ^3^CREATIS, CNRS (UMR 5220), INSERM (U1044), INSA Lyon, University of Lyon, Lyon, France; ^4^Physical Medicine and Sport Traumatology Department, SportS^2^, IOC Research Centre for Prevention of Injury and Protection of Athlete Health, FIFA Medical Centre of Excellence, FIMS Collaborative Centre of Sports Medicine, University Hospital of Liège, University of Liège, Liège, Belgium; ^5^Centre Hospitalier Lyon-Sud, Hospices Civils de Lyon, Pierre-Bénite, France; ^6^Biostatistics Department, University Hospital of Liège, Liège, Belgium; ^7^Neurology Department, Valle d'Aosta Regional Hospital, Aosta, Italy; ^8^Department of Anesthesiology, University Hospital of Saint Etienne, Saint-Étienne, France; ^9^Institute of Sport Sciences, University of Lausanne, Lausanne, Switzerland

**Keywords:** cardiac biomarker, cardiac fibrosis markers, ultramarathon running, ST2, galectin-3

## Abstract

**Objectives:**

The effects of ultra-distance on cardiac remodeling and fibrosis are unclear. Moreover, there are no data reporting the kinetics of cardiac alterations throughout the event and during recovery. Our aim was to investigate the kinetics of biological markers including new cardiac fibrosis biomarkers suppression of tumorigenicity 2 (ST2) and galectin-3 (Gal-3) during and after an extreme mountain ultramarathon.

**Methods:**

Fifty experienced runners participating in one of the most challenging mountain ultramarathons (330 km, D+ 25,000 m) were enrolled in our study. Blood samples were collected at four time points: before (Pre-), at 148 km (Mid-), at the finish line (Post-), and 3 days after the recovery period (Recov-).

**Results:**

The cardiac fibrosis biomarkers (ST2 and Gal-3) increased from Pre- to Mid-. During the second half, ST2 remained higher than pre-values as opposed to Gal-3. Necrosis, ischemia, and myocyte injury biomarkers increased until Mid- then decreased but remained higher at Recov- than Pre-values. Oxidative stress appeared at Mid-. Lipid peroxides remained higher at Recov- compared to Pre-. The maximal value in most of these biomarkers was observed at Mid- and not at Post-.

**Conclusions:**

The present study supports biphasic kinetics of cardiac fibrosis biomarkers, with a relative recovery during the second half of the event that seems specific to this extreme event. Overall, performing at such an extreme ultramarathon seems less deleterious for the heart than shorter events.

## Introduction

Cardiovascular incidents during physical activity known as “sudden cardiac death” (SCD) (1) are rare but can be associated with right ventricular dysfunction, arrhythmia, or dysplasia (2, 3). Blood biochemical markers play a particular role in the diagnosis of several cardiovascular diseases and the stratification of risk and treatment (4). It is assumed that if at rest under normal conditions, the values of these biomarkers are higher than the cut-off limits; then, the subject presents an acute risk of developing cardiovascular disease (4).

A non-optimal level of physical activity is among the SCD risk factors for athletes. There is vigorous debate on a potential ceiling, where above a maximal volume of exercise, there is an increased incidence of SCD (5, 6). One way to explore how extreme exercise loads may affect SCD is to assess ultra-endurance athletes (7). Indeed, in recent years, ultra-endurance events have become increasingly popular (8), and so, there is an emerging clinical urgency to clarify any relationship between exercise volume and SCD.

Several biomarkers such as cardiac troponins, myoglobin, creatine kinase, and natriuretic peptides have been identified as markers of cardiomyocyte damage and stretch (9). Mild to moderate elevations in these markers have been reported following prolonged running exercises (10). It has been reported in extreme mountain ultramarathons (MUM) that athletes experienced heart rate adaptation and myocardial strain in the pre- to mid-race segment, attributable to an increase in extracellular water and subsequent plasma volume due to inflammation (11, 12). Of note, the observed cardiac changes were resolved post-race (11, 12). Less trained athletes might exhibit a higher cardiac risk compared to well-trained runners (13–15). It has been shown that in the long term, some endurance athletes have an increased prevalence of coronary artery disease, myocardial fibrosis, and arrhythmias (16). Moreover, it is unexplored if remodeling and cardiac fibrosis occur during MUM, despite myocardial fibrosis possibly being the cause of arrhythmia and consequently, of sudden death (17) in endurance athletes.

For this purpose, from a biochemical point of view, two emergent biomarkers are of high interest: suppression of tumorigenicity 2 (ST2) and galectin-3 (Gal-3) which are recognized as remodeling and cardiac fibrosis markers (18, 19).

The aim of the present study was therefore to measure the kinetics of most of the available biological markers including the new cardiac fibrosis biomarkers ST2 and Gal-3 during and after an extreme MUM.

## Materials and Methods

The Tor des Geants® (TDG) is a 330 km long MUM, with considerable positive/negative elevation changes (+24,000 m) in the Valley of Aosta (Italy). It is considered one of the most difficult MUM in the world. The altitude along the course ranges from 322 to 3,300 m, including 25 mountain passes over 2,000 m. The maximum time allowed to complete the race is 150 h and the best performance was 71 h 49 min.

The subjects were registered runners recruited in the present study by the race organizers through the mail and public announcements. Informed written consent was provided by each participant. The experimental design of the study was approved by the local ethical committee of the Azienda Regionale Sanitaria USL della Valle d'Aosta (n°900-18/08/2014).

The exercise intensity was assessed by calculating the flat-equivalent speed calculated with the distance as follows (20): flat-equivalent distance = distance (km) + positive elevation change (m)/100.

### Blood Biomarkers Collection and Analysis

The experimental design was a longitudinal study with repeated assessments at four key time points before, during, and after the race. The first session (Pre-) was performed 4 days prior to the race; the second (Mid-), was during the race at mid-point (148.7 km, D+ 9,270 m). At the end of the race, the athletes were evaluated for a third time (Post-) in <1 h after their arrival. The last session was finally performed after 72 h of recovery (Recov-). Bodyweight (kg) and body temperature (°C) were recorded at each session. Body mass index (BMI) was calculated.

Blood samples were collected at each session within 10 mins after arrival at each key point. Samples were drawn from an antecubital vein into a dry, heparinized, and EDTA tube according to the analysis to be performed. Both tubes were immediately centrifuged for 10 mins (3,500 RPM). Since it was not possible to carry out the analyses on the same day by point-of-care technologies, the plasma and serum were frozen at −80°C.

The hematology parameters were directly analyzed by a pocH-100i™ analyzer (Sysmex, Nordstedt, Germany). Cobas 8000 and Cobas 6000 (Roche Diagnostics, Manheim, Germany) were used to perform serial determinations for creatine kinase (CK), creatine kinase muscle and brain (MB) isoform (CKMB), troponin T high sensitive (hsTnT), N-terminal pro-hormone of natriuretic peptides (NT-proBNP), myoglobin (MYO), heart-fatty acid-binding protein (HFABP) (Randox, Crumlin, United Kingdom), C-reactive protein high sensitive (hsCRP), electrolytes [sodium (Na), potassium (K), chloride (Cl)], total proteins (PT), renal biomarkers [serum creatinine (CR), urea (URS), and urinary creatine (CRU)], and oxidized and reduced glutathione (Bioxytech, Burlingame, CA, United States) (GOX and GSH). For Gal-3 measurements, we used an enzyme-linked fluorescent assay (Biomerieux, Marcy l'Etoile, France). Soluble ST2 was measured using a high sensitivity sandwich monoclonal immunoassay (Presage ST2 Assay Critical Diagnostic, San Diego, CA, United States). Lipid peroxides (POXL) and myeloperoxydase (MPO) were measured on Etimax (Diasorin, Saluggia, Itlay) with (Mercodia, Uppsala, Sweden) ELISA kit.

### Statistical Analysis

Data are presented as mean ± SD at each sampling time for biomarkers. Some parameters were log-transformed to normalize their distribution. The generalized linear mixed model was used to test the level of each biomarker over time. Pairwise comparisons between time points were done with the Scheffe *post-hoc* test. The deltas were tested by the paired Student *t*-test. Results were considered significant at the 5% critical level (*p* < 0.05). Data analysis was carried out with Statistical Analysis System (version 9.4 for Windows).

## Results

### Baseline Parameters

A total of 50 runners participated in the Pre- measurements. None of the subjects had clinical or anamnestic evidence of cardiac diseases or hypertension. Nineteen runners withdrew from the race due to different health problems such as diarrhea, fever, or traumatic injuries and were, therefore, not included in the analysis. Twenty-seven runners participated in all the blood-drawn tests. The characteristics of these subjects are displayed in Table 1. The mean finishing time of our subjects was 126 h 01 min 44 s ± 14 h 05 min 21 s. The flat-equivalent speed was 5.7 ± 0.9 km/h for the first segment and 3.7 ± 0.4 km/h for the second half, corresponding to a 33 ± 8% difference.

**Table 1:** Demographic and training profile data.

	**Pre**	**Mid**	**Post**	**Recov**
N	50	32	32	29
Sex (male/female)	46/4	31/1	31/1	29/0
Age (years)	43.0 ± 9.1	43 ± 8.6	43 ± 8.6	43.1 ± 8.3
Height (m)	1.75 ± 6.20	1.75 ± 6.40	1.75 ± 6.40	1.75 ± 5.60
Weight (kg)	72.2 ± 8.0	71.7 ± 8.2	71.7 ± 8.2	70.8 ± 7.3
BMI (kg m^−2^)	23.6 ± 2.0	23.4 ± 2.0	23.4 ± 2.0	23.1 ± 2.0
Body temperature (°C)	36.2 ± 0.9	37.3 ± 0.5	37.3 ± 0.5	37.1 ± 0.7
Pain		4.1 ± 2.9	4.1 ± 2.9	1.08 ± 1.7
Training (hours of running)/week (n)	3.9 ± 1.7	3.9 ± 1.5	3.9 ± 1.5	4.0 ± 1.4
Running experience (years)	14.2 ± 10.4	13.5 ± 10.5	13.5 ± 10.5	13.1 ± 9.8
Experience in ultra-marathons (years)	5.3 ± 3.6	5.5 ± 3.6	5.5 ± 3.6	5.3 ± 3.6
Previous ultra-marathons (years)	13.0 ± 10.0	11.0 ± 9.0	11.0 ± 9.0	11.5 ± 9.0

### Biological Analysis

We checked the impact of the hematocrit (HCT) on all the biomarkers and the ratio was maximum between Pre- and Post- of 1.13. This means that there is only a 13% difference between results corrected or not by hematocrit. All biological data are shown in Table 2. Pre-levels were within the reference values range, i.e., considered as normal for a healthy population. Significant increases were observed especially at Mid.

**Table 2:** Blood biomarker concentrations (mean ± SD) at different time points and the *p*-value in the Tors de Geants (TDG) runners.

		**Time points**				***P*** **-values**			
	**References values**	**Pre-**	**Mid-**	**Post-**	**Recov-**	***P*-value**	***P*-value**	***P*-value**	***P*-value**
**Variable (units)**		**Mean ±SD**	**Mean ±SD**	**Mean ±SD**	**Mean ±SD**	**Overall**	**TPre-TMid**	**TMid-Tpost**	**TPost-Trecov**
**Remodelling-fibrosis**									
ST2 (ng/ml)	<35	28 ± 15.4	78.9 ± 40.7	61 ± 32.5	28.9 ± 11.4	<0.0001	<0.0001^*^	0.047^**^	<0.0001^**^
Galectine-3 (ng/ml)	<17.9	9 ± 1.8	11 ± 2.3	10.1 ± 2.1	9.1 ± 1.5	<0.0001	<0.0001^*^	0.012^**^	0.0015^**^
**Ischemia-necrosis**									
Creatine kinase (UI/L)	30–175	135 ± 72.1	9,016 ± 9,834	3,276 ± 3,353	675 ± 530	<0.0001	<0.0001^*^	<0.0001^**^	<0.0001^**^
Creatine kinase MB (μg/L)	0–6	3.1 ± 2.2	84.7 ± 63.6	45.6 ± 33.8	13.1 ± 7.2	<0.0001	<0.0001^*^	<0.0001^**^	<0.0001^**^
CKMB/CK (μg/100UI)	0–5	2.3 ± 0.8	1.2 ± 0.6	2 ± 1.5	2.4 ± 1.3	<0.0001	<0.0001^**^	<0.0001^*^	0.018^*^
Heart fatty acid binding protein (ng/ml)	<3.55	6.8 ± 5.2	76.2 ± 75.7	32.9 ± 24.3	8.6 ± 4.3	<0.0001	<0.0001^*^	0.0057^**^	<0.0001^**^
Myoglobin (μg/L)	28–72	28 ± 8.6	1,207 ± 1,287	441 ± 384	80.1 ± 43.3	<0.0001	<0.0001^*^	<0.0001^**^	<0.0001^**^
hsTnT (ng/L)	<14	5.5 ± 1.1	12.7 ± 7	9.7 ± 4.3	6.9 ± 2	<0.0001	<0.0001^*^	0.069	0.0032^**^
Copeptine (pmol/L)	<12	5.9 ± 2.9	20.7 ± 12.2	13.7 ± 8.6	7.9 ± 3.8	<0.0001	<0.0001^*^	0.0029^**^	0.0004^**^
**Inflammation**									
White blood cell (10^3^/mm3)	3.6–15	7 ± 1.8	10.5 ± 2.2	8.1 ± 2	6.4 ± 1.4	<0.0001	<0.0001^*^	<0.0001^**^	<0.0001^**^
Total protein (g/L)	62–78	72.7 ± 3.6	68.1 ± 4	65.7 ± 4.7	66.2 ± 4.0	<0.0001	<0.0001^**^	0.019	0.78
CRP (mg/L)	0–6	1.2 ± 1.9	18.2 ± 12.3	13.7 ± 12.9	5.7 ± 4.3	<0.0001	<0.0001^*^	0.062	<0.0001^**^
**Oxidative stress**									
Oxidized glutathion (μmol/L)	<10	1.6 ± 1.2	6.2 ± 8.4	3.2 ± 8	6.6 ± 14.1	0.016	0.064	0.22	0.34
Reduced glutathion (μmol/L)	717–1110	811 ± 105	742 ± 80.8	745 ± 89.3	746 ± 106	0.0025	0.015^**^	0.99	1
Ratio total glutathion/oxidized glutathion	111–747	628 ± 221	443 ± 294	547 ± 231	446 ± 286	0.0077	0.028^**^	0.39	0.47
Myeloperoxdase (ng/ml)	<55	34.8 ± 16.8	35.5 ± 17.1	30.9 ± 15.2	31.3 ± 13.8	0.0005	0.96	0.007^**^	0.99
Lipid peroxides (μmol/L)	<432	273 ± 155	311 ± 175	528 ± 217	638 ± 184	<0.0001	0.79	<0.0001^*^	0.015^*^
**Renal function**									
Creatinine (mg/dl)	0.72–1.18	1 ± 0.1	1.1 ± 0.1	1 ± 0.1	0.9 ± 0.1	<0.0001	0.002^*^	0.0055^**^	0.0004^**^
Urinary creatinine (g/L)	0.24–2.55	1.3 ± 0.8	1.9 ± 0.8	1.7 ± 0.7	1 ± 0.5	<0.0001	0.015^*^	0.72	0.002^**^
Uric acid (mg/L)	<70	5.2 ± 0.9	5.8 ± 1.3	5.4 ± 1.5	4.3 ± 0.8	<0.0001	0.014^*^	0.11	<0.0001^**^
Urea (g/L)	16–48	36.4 ± 7.9	66.8 ± 19.1	49.5 ± 13.3	35.1 ± 7.4	<0.0001	<0.0001^*^	<0.0001^**^	<0.0001^**^
**Myocyte-stress biomarkers**									
NT-proBNP (ng/L)	<103	50.3 ± 1.4	472 ± 383	396 ± 374	95.5 ± 59.4	<0.0001	<0.0001^*^	0.41	<0.0001^**^
**Ions**									
Potassium (mmol/L)	3.5–5.1	4.1 ± 0.4	3.7 ± 0.3	3.6 ± 0.5	3.9 ± 0.4	<0.0001	0.0008^**^	0.84	0.031^*^
Sodium (mmol/L)	135–145	141 ± 1.8	141 ± 2.6	140 ± 3.2	141 ± 1.9	0.32	0.99	0.87	0.33
Chlore (mmol/L)	98–107	101 ± 1.7	103 ± 2.8	103 ± 2.9	104 ± 2.9	<0.0001	0.0009^*^	0.99	0.50

* *Significative increase*.

** *Significative decrease*.

### Cardiac Fibrosis Biomarkers

Galectin 3 (Gal-3) increased by 39 ± 25% from Pre- to Mid-, then increased slowly to Post- and returned to baseline values at Recov. As opposed to Gal-3, ST2 increased from more than 270 ± 230% from Pre- to Mid- but decreased from Mid- to Post- but remained elevated at Recov- (27 ± 46% higher than Pre-values).

### Necrosis-Ischemia Biomarkers

As expected, we found a significant increase for hsTnT (165 ± 136%), HFABP (1,815 ± 2,245%), CK (8,760 ± 9,323%), CK-MB (3,890 ± 3,982%), and MYO (5,203 ± 6,128%) from Pre- to Mid-. During the second half of the race, these biomarkers decreased from Mid- to Post- but remained higher at Recov- than the baselines values: hsTnT (40 ± 54%), HFABP (1.0 ± 1.3%), CK (493 ± 501%), CK-MB (418 ± 313%), and MYO (231 ± 181%).

### Myocytes-Stress Injury Biomarkers

NT-proBNP increased significantly, with a rise of 1,003 ± 847% between Pre- and Mid-, remained at the same level up to Post-, but decreased (−64 ± 32%) during the recovery (109 ± 134% higher than Pre-).

### Oxidative Stress Biomarkers

Reduced glutathione decreased by 8.1 ± 9.7% between Pre- and Mid- and stayed stable thereafter. The ratio of total glutathione/oxidized glutathione decreased significantly by 126 ± 412% between Pre- and Mid- before an increase of 186 ± 437% at Post- and finally came back at Recov- to the same values at Mid-. MPO remained stable at Mid- before decreasing. POXL increased slowly from Pre- at 26.8 ± 68.0% to be maximum at Recov- and remained higher at 182 ± 122% than at Pre-.

### Inflammation

C-reactive protein high sensitive (hsCRP) increased by 2,717 ± 1,980% from Pre- to Mid-, remained high between Mid- and Post- and then decreased from Post- to Recov- but remained higher than the baseline values (795 ± 840% higher than the Pre- values). White blood cells increased from Pre- to Mid- (80 ± 54%) but then decreased and returned to baseline values at Recov-.

### Renal Functions Biomarkers

Plasmatic CR and URS (urea) levels increased by 24 ± 14% and by 89 ± 50%, respectively, during the Pre- to Mid-phase before decreasing and returning to baseline values at Recov-.

## Discussion

Over the last decade, there is an increasing interest in the cardiac and muscular disorders induced by ultra-endurance events. The novelty of the present study is the display of the changes of many biomarkers not only after the race (Post-) but during (Mid-) and after 3 days of recovery (Recov-), which add great value to the understanding of the underlying mechanisms because the kinetics of release is different for each biomarker. In previous studies, sample withdrawal was limited to immediate post-race or, at most, within 24 h post-exercise, most likely because of practical difficulties in the sample collection for longer recovery periods (21). Some studies have taken blood 24-, 48-, or sometimes 72 h or more after the event but concentrated their discussion on the increase observed just after the race and did not discuss that this was related to after the acute recovery phase (15, 22–24). Moreover, while most of the published studies have focused principally on the release of cTroponins and natriuretic peptides (25–27), we have included an analysis of two emerging biomarkers, ST2 and Gal-3, known as remodeling and cardiac fibrosis markers (15, 18). Overall, we observed a transient biphasic increase in different cardiac and inflammatory biomarkers, however, the increase was often lower than that observed for shorter races.

The highest exercise intensity exerted by the athletes was during the first segment of the race which may have influenced the initial increase in muscle, inflammatory, and cardiac biomarkers measured at Mid-. After the mid-point, lower susceptibility to skeletal muscle damage because of repeated bouts of the same exercise (28) may be an explanation for the increasing level of most biomarkers that we tested. Furthermore, the athletes ran at a slower pace after Mid- most likely because of the accumulation of fatigue. After the recovery period, remodeling and fibrosis, ischemia-necrosis and inflammatory biomarkers, and the huge rise observed at Mid-, had a trend to return to the baseline values with a rate according to their half-life. The 33% lower intensity exerted by the athletes during the second half of the race, is in line with previous results (6.2 ± 2.1 and 4.5 ± 0.4 km/h) reported for the same event in Saugy et al. (20) and indicates that most of the participants were only walking slowly during the final part. A similar decrease in intensity was also shown by the changes in vertical speed (m/h) measured during the uphill portion of the mountain passes throughout the race (29). The biphasic responses reported in the present study were not limited to biomarkers since we already showed a similar biphasic time course (i.e., larger at mid-race than at the end) for cardiac fatigue (11) or alterations in postural control (30).

We believe that two mechanisms (i.e., the large decrease in exercise intensity and the large increase in extracellular water) are specific to this extreme MUM and may explain the biphasic pattern in the physiological responses tested. Of interest is that multi-stage ultra-endurance road running events, such as the TransEurope Footrace (4,500 km in 64 days) induce a relatively stable speed across stages and consequently different kinetics of biomarkers than in the present MUM (31). Something very interesting to highlight is the similar response of the different biomarkers despite their different molecular size, which commonly influences these responses during exercise (21, 32).

### Cardiac Remodeling-Fibrosis Biomarkers

Studies have focused on the main fibrosis cardiac biomarkers in relation to endurance events (15, 25, 33–35). In the present study, we included two emerging biomarkers, ST2 and Gal-3. Notably, we observed a large and transient change for ST2, while there was a smaller increase in Gal-3.

### ST2

During an extreme running event, even in trained athletes, the ST2 complex likely reflects a high degree of stress, i.e., the highest concentration of 163 ng/ml is observed at Post- and returns to baseline values during the recovery.

If ST2 remains elevated, further examination is recommended (possibly due to exacerbating underlying vulnerability, such as genetic cardiomyopathies, subclinical cardiac ischemia) to determine the origin of this sustained elevation (36, 37).

Since every 10 ng/ml increase in ST2 was associated with a ~20% increase in cardiovascular risk (38), the increase of the ST2 during MUM could reflect, if repeated regularly, the remodeling and development of cardiac fibrosis, as shown previously for increased stress or injury to the myocardium due to acute myocardial infarction, uncontrolled hypertension, and other forms of myocyte damage. Indeed, replacement fibrosis appears at sites of previous cardiomyocyte necrosis but not without adverse functional consequences such as increased vulnerability for arrhythmias, impact on systolic and diastolic function (39). Fibrosis is a fundamental component of the adverse structural remodeling of the myocardium present in the failing heart (39). It is well known that responses to acute and chronic damage can involve recruitment of immune cells to the myocardium; production of cell signaling proteins from mast cells and macrophages, resulting in activation of fibroblasts and myofibroblasts; and the deposition of procollagen into the extracellular matrix, which is irreversibly cross-linked to collagen-generating cardiac fibrosis (38, 40).

### Gal-3

Reportedly, Gal-3 is not a dynamic biomarker that could explain different kinetics, when compared to ST2 (18). However, previous authors have shown plasma Gal-3 is substantially elevated in endurance athletes after running although no correlation with cardiac function or myocardial fibrosis (25). Contradicting these findings, other studies have shown that increased Gal-3 was associated with biochemical abnormalities during high-intensity endurance exercise, which may reflect adverse consequences on cardiac structure (33). It is possible that the discordance rise is a consequence of the training status of the athletes, a kind of physiological adaptation, in line with one of our previous studies with marathon runners (41).

Note that despite the emergence of these markers as indicators of fibrosis and cardiac remodeling, they have not been associated with clinical cardiac fibrosis reported with MRI in athletes.

### Other Biomarkers

A novel finding is that most of these biomarkers (see Table 2) reached their maximal values at Mid-, displaying a biphasic pattern that is similar to what has been previously reported for cardiac fatigue (30) and postural control (30). This biphasic response seems specific to this type of extreme MUM as the pattern is not for shorter endurance events (e.g., marathon, triathlon). This is most likely because it takes a certain amount of time for the release of the biomarkers into the blood to levels that can be detected. During the shorter distance races, the maximum release may not be observable at the mid-point of the race.

Many mechanisms may explain the initial rise of biomarkers (Pre- to Mid-) including myocardial damage due to mechanical stretch or increased membrane permeability, leading to the increase in troponins, CK, HFABP. Moreover, hypoxia activates the neuro-endocrine system, leading to hemodynamic modification (overload and/or impaired left ventricular relaxation) and consequently to the over-expression of NT-proBNP (33). Similar increases to that observed in the initial (Pre- to Mid-) section of the present MUM have been observed in marathons for the NT-proBNP (42–45). Moreover, it is possible that a physiological remodeling of the myocardium as an adaptation to exercise could explain why athletes have bigger hearts with smaller troponin increase post-exercise (38–40).

The decrease or plateau in most of these biomarkers during the second half (Mid- to Post-) of the MUM, despite accumulating fatigue, is likely caused by the low exercise intensity shown by the running velocity (11), consequence of the combination of extreme distance and sleep deprivation. The mean intensity during the TDG was measured as ~50 % of VO_2_max (46), much lower than the 65–85% measured on shorter distances such as the marathon. Moreover, the intensity during this MUM was estimated to be 20–25% lower in the second half than in the first (20). This leads to specific hemodynamic changes, specific kinetics in HR and to transient cardiac dysfunction that was observed at Mid- but not at Post- (11).

For the inflammatory responses, we observed that the highest increases in serum hsCRP concentration coincided with the highest levels of serum protein observed at Mid- followed by a slow decrease. For the estimation of the renal function, Cr, URS, and GFR were used. We observed only increases of Cr and URS between Pre- and Mid- but these were not clinically significant as to describe a renal failure. The concentration of Cr did not meet the risk criteria for acute kidney injury, as previously described over a shorter (200 km) MUM (47). Muscle damage rarely causes adverse consequences for athletes during a MUM. However, the release of excessive amounts of intramuscular proteins into circulation can negatively affect renal function mainly in conditions of heat, dehydration, body mass decrease, or use of non-steroidal anti-inflammatory drugs during the race (48, 49). The impact of the hydration was negligible as the maximal value of the HCT ratio between Pre- and the other times was 1.13, so it was not clinically significant according to the biological variation of the different biomarkers.

### Oxidative Stress Biomarkers

The available scientific literature describes the effects of high-intensity exercise on the increase of oxygen consumption and production of its reactive forms (50–52). In the current study, the most significant result for oxidative stress is the increase in lipid peroxide. Literature is scarce about lipid peroxidation in ultra-endurance sports but oxidation of HDL and decrease of oxidation of LDL have been documented (53). The POXL increase could be explained by the increase of oxidized HDL (53), induced by exercise, accelerating the transport of lipid oxidation products by HDL (53, 54). For the other stress biomarkers, we did not observe any significant change, suggesting that the athletes had training-induced adaptation (55).

### Individual Approach

The individual approach is very interesting. Indeed, if we have a closer look at Mid- for different subjects, we can observe that one subject showed a CK concentration of 34,874 UI/L with a CKMB concentration of 228 UI/L and an NT-proBNP of very high concentration 2,202 ng/L. Three other subjects also had very high concentrations of CK >23,000 UI/L with high CKMB also correlated with an NT-proBNP around 1,200 ng/L. Of note, it was not the same subject who showed the high hsTnT. So, it appears that there is no link between CK increase and TnThs. In this subject, only one subject had a CKMB/CK ratio >4% with a CK of 961 UI/L and a CKMB of 31 UI/L suggestive of cardiac injury. However, his hsTnT concentration of 8 ng/L was not very elevated. Interestingly, ST2 and NT-proBNP of this subject were low: 33 ng/ml and 87 ng/L, respectively.

This means that different mechanisms are involved. The subjects with high ST2 and NT-proBNP concentrations probably suffered from a myocyte stress phenomenon and perhaps the onset of cardiac remodeling, whereas the subject with the highest troponin concentration probably suffered from at least transient ischemia.

The highest increase observed for ST2 was associated with a high level of CK but was not observed for the subject showing the highest increase of CK and NT-proBNP. ST2 increase seems independent of these other cardiac damage markers. Finally, we observed a link between increased CK and hsCRP. Indeed, the highest hsCRP increase was observed for the subjects with the highest increase of CK, proof of muscle damage, and subsequent inflammation.

Furthermore, there might be many different factors that influence the marked variation among individuals in the release of exercise-associated cardiac biomarkers (14, 56–58). Keep in mind that analytical variation remained relatively moderate as we group measured all the samples. However, one could not eliminate the inevitable biological variation, which must be considered for the interpretation of results. We observed high inter-subject variability in all biochemical factors despite similar mean age and BMI. The relationship between exercise, damage/adaptation, and biomarker fluctuation is very complex, due to a number of variables involved, including the type of sport practiced, intensity, duration of exercise, and training (59). However, our results are strengthened by the homogeneity between subjects with similar training loads and running experience. In the present study, each participant was his own control to reduce the bias.

A limitation of the study is that Mid-point does not coincide with the maximum value of one of the markers, such as troponin. It was impossible to draw blood more often and therefore the peak may have occurred earlier during the race. This may explain why the values were lower than those observed in marathons, for example. Another explanation would be the slower pace achieved by the athletes, due to the much longer distance of the MUM (60).

## Conclusions

The clinical significance of changes in cardiac biomarkers is debated. By investigating the kinetics of these biomarkers during an extreme MUM known to induce larger cardiac fatigue during the first half than the second half of the event, one may report their kinetics and test their significance. Our conclusions support the biphasic kinetics (i.e., most of the cardiac biomarkers being higher at Mid- than at Post-) that seemed specific to this type of event. Of importance is that the ST2 and Gal-3 (markers of fibrosis and cardiac remodeling) changes are in line with the assumption that MUM may be less deleterious than shorter and more intense endurance events. In further studies, these markers of cardiac fibrosis may be used to highlight the risk to develop myocardial fibrosis (already described in endurance athletes) and correlated to MRI observations in order to predict myocardial fibrosis development.

## Data Availability Statement

The raw data supporting the conclusions of this article will be made available by the authors, without undue reservation.

## Ethics Statement

The studies involving human participants were reviewed and approved by Comité d'Éthique Hospitalo-Facultaire Universitaire de Liège. The patients/participants provided their written informed consent to participate in this study.

## Author Contributions

CL, EC, GM, J-FK, PC, and MV contributed to conception and design of the study. CL organized the database and wrote the first draft of the manuscript. CL and LS performed the statistical analysis. All authors contributed to manuscript revision, read, and approved the submitted version.

## Funding

The Leon Fredericq foundation supported this study with an operational grant.

## Conflict of Interest

The authors declare that the research was conducted in the absence of any commercial or financial relationships that could be construed as a potential conflict of interest.

## Publisher's Note

All claims expressed in this article are solely those of the authors and do not necessarily represent those of their affiliated organizations, or those of the publisher, the editors and the reviewers. Any product that may be evaluated in this article, or claim that may be made by its manufacturer, is not guaranteed or endorsed by the publisher.
